# Brazilian initial experience with lung transplantation due to irreversible lung fibrosis post-COVID-19 in a national reference center: a cohort study

**DOI:** 10.1590/1516-3180.2021.0842.R1.13102021

**Published:** 2021-12-17

**Authors:** Flavio Pola dos Reis, Lucas Matos Fernandes, Luis Gustavo Abdalla, Silvia Vidal Campos, Priscila Cilene Leon Bueno de Camargo, Samuel Lucas dos Santos, Ivana Teixeira de Aguiar, Juliana Patricia Pires, Andre Nathan Costa, Rafael Medeiros Carraro, Ricardo Henrique de Oliveira Braga Teixeira, Paulo Manuel Pêgo-Fernandes

**Affiliations:** I MD. Attending Surgeon, Thoracic Surgery Program, Instituto do Coracao, Hospital das Clinicas HCFMUSP, Faculdade de Medicina, Universidade de Sao Paulo, Sao Paulo, SP, BR.; II MD, PhD. Attending Surgeon, Thoracic Surgery Program, Instituto do Coracao, Hospital das Clinicas HCFMUSP, Faculdade de Medicina, Universidade de Sao Paulo, Sao Paulo, SP, BR.; III MD. Attending Surgeon, Thoracic Surgery Program, Instituto do Coracao, Hospital das Clinicas HCFMUSP, Faculdade de Medicina, Universidade de Sao Paulo, Sao Paulo, SP, BR.; IV MD. Attending Physician, Division of Pulmonology, Instituto do Coracao, Hospital das Clinicas HCFMUSP, Faculdade de Medicina, Universidade de Sao Paulo, Sao Paulo, SP, BR.; V MD, PhD. Attending Physician, Division of Pulmonology, Instituto do Coracao, Hospital das Clinicas HCFMUSP, Faculdade de Medicina, Universidade de Sao Paulo, Sao Paulo, SP, BR.; VI MD. Lung Transplant Resident, Thoracic Surgery Program, Instituto do Coracao, Hospital das Clinicas HCFMUSP, Faculdade de Medicina, Universidade de Sao Paulo, Sao Paulo, SP, BR.; VII MD. Lung Transplant Fellow, Thoracic Surgery Program, Instituto do Coracao, Hospital das Clinicas HCFMUSP, Faculdade de Medicina, Universidade de Sao Paulo, Sao Paulo, SP, BR.; VIII MD. Lung Transplant Fellow. Division of Pulmonology, Instituto do Coracao, Hospital das Clinicas HCFMUSP, Faculdade de Medicina, Universidade de Sao Paulo, Sao Paulo, SP, BR.; IX MD, PhD. Attending Physician, Division of Pulmonology, Instituto do Coracao, Hospital das Clinicas HCFMUSP, Faculdade de Medicina, Universidade de Sao Paulo, Sao Paulo, SP, BR.; X MD. Attending Physician, Division of Pulmonology, Instituto do Coracao, Hospital das Clinicas HCFMUSP, Faculdade de Medicina, Universidade de Sao Paulo, Sao Paulo, SP, BR.; XI MD, PhD. Attending Physician, Division of Pulmonology, Instituto do Coracao, Hospital das Clinicas HCFMUSP, Faculdade de Medicina, Universidade de Sao Paulo, Sao Paulo, SP, BR.; XII MD, PhD. Full Professor, Thoracic Surgery Program, Instituto do Coracao, Hospital das Clinicas HCFMUSP, Faculdade de Medicina, Universidade de Sao Paulo, Sao Paulo, SP, BR; Director, Scientific Department, Associação Paulista de Medicina, São Paulo (SP), Brazil.

**Keywords:** Extracorporeal membrane oxygenation, Lung transplantation, COVID-19, ECMO, Lung transplant, End-stage lung disease

## Abstract

**BACKGROUND:**

Lung transplantation (LTx) has been discussed as an option for treating irreversible lung fibrosis post-coronavirus disease 2019 (COVID-19), in selected cases.

**OBJECTIVES:**

To report on the initial experience and management of end-stage lung disease due to COVID-19 at a national center reference in Brazil.

**DESIGN AND SETTING:**

Cohort study conducted at a national reference center for lung transplantation.

**METHODS:**

Medical charts were reviewed regarding patients’ demographics and pre-COVID-19 characteristics, post-LTx due to COVID-19.

**RESULTS:**

Between March 2020 and September 2021, there were 33 cases of LTx. During this period, we evaluated 11 cases of severe COVID-19-related acute respiratory distress syndrome (ARDS) that were potentially candidates for LTx. Among these, LTx was only indicated for three patients (9.1%). All of these patients were on venovenous extracorporeal membrane oxygenation (ECMO), and the procedure that they underwent was central venoarterial ECMO. All three patients were still alive after the first 30 postoperative days. However, patient #1 and patient #2 subsequently died due to fungal sepsis on the 47^th^ and 52^nd^ postoperative days, respectively. Patient #3 was discharged on the 30^th^ postoperative day.

**CONCLUSIONS:**

LTx is feasible among these complex patients. Survival over the first 30 days was 100%, and this favors surgical feasibility. Nonetheless, these were critically ill patients.

## INTRODUCTION

Coronavirus disease 2019 (COVID-19), caused by the severe acute respiratory syndrome coronavirus 2 (SARS-CoV-2), is an infectious disease with potentially severe manifestations.^1^ Most patients with COVID-19 have a mild or asymptomatic disease course; however, about 10% require admission to an intensive care unit (ICU) because of acute respiratory distress syndrome (ARDS).^2^ Mortality rates of up to 60% have been reported for this subgroup^3^ but, in Brazil, this rate rises to 80%.^4^

Much has been discussed about mechanical ventilation, neuromuscular blockade and prone and extracorporeal membrane oxygenation for ventilatory support^5^^,^^6^^,^^7^ for these patients. However, some patients evolve with severe lung fibrosis.^8^ For this group of patients, lung transplantation (LTx) has been discussed as a treatment option. The first reports of lung transplantation post-COVID-19 were in 2020, first from China^9^ and then from Austria.^10^ Lung transplantation is potentially lifesaving, but the true effect of the procedure in the acute setting of COVID-19 needs to be very well discussed because these patients are critically ill, with a long hospital stay and associated morbidity.^11^

## OBJECTIVE

The objective of this article was to report on the initial experience of LTx for management of end-stage lung disease due to Covid-19 at a national reference center in Brazil.

## METHODS

### Study design

This cohort study was conducted at the Heart Institute of Hospital das Clínicas (HC), Faculdade de Medicina da Universidade de São Paulo (FMUSP). It is the largest academic, tertiary-level, university-affiliated hospital in Brazil, with 300 adult ICU beds that were dedicated exclusively to caring for COVID-19 patients during the peak of the pandemic.^1^ Our institution is also the reference center for ARDS/extracorporeal membrane oxygenation (ECMO) and LTx of the state of São Paulo. This project was approved by our institutional review board under the number CAAE 51617821.2.0000.0068, on October 1, 2021.

Firstly, we entered discussions to determine criteria for evaluating patients with ARDS in association with COVID-19, in a Technical Committee for Thoracic Organ Transplantation for the state of São Paulo, jointly with the three centers that perform lung transplantation in this state. The protocol thus elaborated was then ratified by the Ministry of Health, in a National Technical Committee (Appendix 1). We used references published by Cypel and Keshavjee^12^ and the guidance from the International Society of Heart and Lung Transplantation regarding the SARS CoV-2 pandemic (revised February 2021), including our local particularities.

### Data collection

Patients’ medical charts were reviewed regarding demographic and pre-COVID-19 characteristics, including comorbidities, pre-transplantation profile (including clinical and radiological features, treatment and management, medical course and indications for lung transplantation), perioperative challenges (including transfusion requirements and technical challenges), pathological assessment of the explanted lungs and post-transplantation outcomes. The characteristics of lung donors were also recorded.

### Surgical technique

The procedure was performed under general anesthesia through a Clamshell incision. The tracheostomy cannula was removed and a selective tube was placed. Its position was checked by means of fibroscopy. The patient was positioned supinely with the head at the midline. The preparation started at the chin and went down to the abdomen. Venovenous (VV) ECMO was maintained, although with reduced flow of 1.5 liters per minute, without oxygenation. Venoarterial (VA) ECMO with central cannulation was chosen instead of cardiocirculatory assistance.

The lung transplantation procedure was sequential and bilateral. During the reperfusion, the cardiopulmonary assistance was reduced to 1.5 liters per minute and arterial clamping was opened. Following this, the atrial clamping was opened and cardiopulmonary assistance was returned to the previous level. At the end of the procedure, a transesophageal echocardiogram was performed to check that the right and left ventricular function had been preserved. If so, we proceeded with weaning off central ECMO. After the thorax had been closed, the blood gas showed a pO_2_/FiO_2_ ratio > 250, after weaning off VV ECMO.

### ECMO installation

Central VA ECMO with right atrial cannulation for venous drainage and aortic return cannulation was used. The circuit configuration consisted of a centrifugal pump with a polymethylpentene oxygenator (Rotaflow/Quadrox-ID, Getinge Cardiopulmonary AG, Hirrlinger, Germany), connected to 3/8” inner diameter tubing with a bridge between arterial venous limbs. The circuit was primed with Plasma-Lyte. Heparin was titrated to keep the activated clotting time (ACT) around 180-250 seconds, and additional blood products were used when necessary. The arterial flow was calculated in accordance with body surface area, with a target of 70% of the nominal flow. It was corrected when necessary based on the hemodynamic and metabolic demand during the procedure, through monitoring the arterial and venous pressures and the circuit pressure. An ECMO specialist controlled the cardiopulmonary support.

## RESULTS

Between March 2020 and September 2021, there were 33 cases of lung transplantations. During this period, we evaluated 11 cases of severe COVID-19-related ARDS that were potentially candidates for LTx. Six cases were evaluated in hospitals within our own complex (HC-FMUSP) and five cases were evaluated by means of telemedicine connections to other national reference centers.

Three of the six cases evaluated in our complex did not proceed further: two patients did not meet the criteria described earlier, i.e. they presented body mass index (BMI) > 35 kg/m^2^ and had social issues; and one patient died from sepsis. The other three patients completed the evaluation and were included in the waiting list. By the time when this paper was written, all of these three patients had undergone LTx.

Only one of the cases evaluated by means of telemedicine met all the criteria (Appendix 1). This patient was transferred to our institution on mechanical ventilation. However, after the initial treatment and rehabilitation, she achieved pulmonary improvement and is now in the ward and not dependent on oxygen. We decided to decline to perform lung transplantation at this time, and she will be referred to the clinic after hospital discharge.

During the pandemic, 33 cases of lung transplantation were performed and three of these (9.1%) were due to irreversible pulmonary fibrosis associated with COVID-19. Before COVID-19, these three patients did not have any comorbidities and had normal lives without addictions. All of these three patients were referred to our hospital for treatment of COVID-19 because we are the reference center for ECMO and LTx. Given the clinical improvement but irreversible lung injury, lung transplantation was proposed. The length of time for which they had been receiving ECMO support at the time of waitlisting for LTx was 83, 52 and 77 days, respectively. All three patients were awake and agreed to undergo LTx. Before the transplantation, their renal, liver and cardiac functions were normal. Their lung allocation scores were 85.751, 85.000 and 82.097 respectively, at the time of waitlisting. Their clinical features are presented in Table 1.

**Table 1.** Patient characteristics and demographics

Patient	# 1	# 2	# 3
Sex	Male	Female	Male
Age (years)	46	34	31
Height (cm)	174	171	167
Weight (kg)	75	85	75
Body mass index	24.77	29.06	26.8
Blood group	A	A	O
Length of time from COVID-19 diagnosis to ICU admission (days)	8	14	5
Length of time from COVID-19 diagnosis to intubation (days)	9	19	6
COVID-19 specific treatment	Steroids	Steroids	Steroids
Tracheostomy	Yes	Yes	Yes
Type of ventilation	PCV	PCV	PCV
Lung compliance (ml/mbar)	8	6	6
ECLS	VV ECMO (two cannulae)	VV ECMO (two cannulae)	VV ECMO (two cannulae)
Length of ECLS support at the time of waitlisting (days)	83	52	77
Awake for ECLS bridging	Yes	Yes	Yes
Recovered from acute kidney injury	No	No	No
Recovered from sepsis	Yes	Yes	Yes
Creatinine (mg/dl)	1.39	0.35	0.45
Blood urea nitrogen (mg/dl)	94	24	36
Aspartate transaminase (U/l)	19	26	30
Alanine aminotransferase (U/l)	23	38	36
International normalized ratio	1,1	1,2	1.1
C-reactive protein (mg/l)	18	22.9	27.5
Leukocytes (total/mm^3^)	9,480	14,000	5,340
Procalcitonin (ng/ml)	-	-	-
Evidence of pulmonary bacterial colonization	*Acinetobacter baumannii* + *Pseudomonas aeruginosa* + *Enterococcus faecalis*	*Acinetobacter baumannii*	*Pseudomonas aeruginosa* R-Carbapenem + *Enterococcus faecalis*
Evidence of fungal colonization	*Candida albicans*	*Candida tropicalis*	
Right ventricular dysfunction	No	No	No
Systolic pulmonary arterial pressure		48	
Time to listing to transplantation (days)	94	76	87
Lung allocation score	85.751	85.000	82.097

ICU = intensive care unit; ECLS = extracorporeal life support; PCV = pressure-controlled ventilation; VV = venovenous; ECMO = extracorporeal membrane oxygenation.

Details of the donors are provided in Table 2. All of them were brain death donors, through traumatic brain injury. They had normal bronchoscopy and pO_2_/FiO_2_ ratios of up to 300.

**Table 2.** Donor features

Patient	# 1	# 2	# 3
Sex	Male	Male	Male
Age (years)	19	34	21
Height (cm)	180	168	170
Weight (kg)	80	72	55
Predicted total lung capacity (liters)	7.14 / 7.30	6.20 / 6.34	5.49 / 5.6
Smoking history (current or past smoker)	Yes	No	Yes
Cause of death	Traumatic brain injury	Traumatic brain injury	Stroke
Chest X-ray	Normal	Normal	Normal
Median intubation time (hours)	72	48	120
PaO_2_/FiO_2_, at time of offer	316	389	355
PaCO_2_, at time of offer (mmHg)	48	33	37
Bronchoscopy	Normal	Normal	Normal
Type of donor	Marginal (chest trauma)	Ideal	Marginal (intubation time)
Median Oto score	5	3	2

The technical features of the operation are presented in the methods section. The lengths of the surgical procedures (skin to skin) were 465, 515 and 550 minutes, respectively. The total durations of ischemia were 345, 275 and 360 minutes, respectively. There was no need for fresh frozen plasma during the procedures (Table 3).

**Table 3.** Transplantation features

Patient	# 1	# 2	# 3
Length of time on the waiting list (days)	8	22	50
Clamshell incision	Yes	Yes	Yes
ECMO intraoperative support	Yes	Yes	Yes
Whole lung transplantation	Yes	Yes	Yes
Surgery time (skin to skin) (min)	465	515	550
Total ischemic time (min)	345	275	360
Number of intraoperative pRBC units	5	5	7

ECMO = extracorporeal membrane oxygenation; pRBC = packed red blood cell.

All the patients were still alive after the first 30 postoperative days. However, patient #1 and patient #2 died due to fungal sepsis on the 47^th^ and 52^nd^ postoperative days, respectively. Patient #3 was discharged on the 30^th^ postoperative day. Patient #2 had primary graft dysfunction at 72 hours (grade 2). No bleeding problems occurred. Patient #1 presented acute cholecystitis and underwent laparoscopic cholecystectomy. All three patients presented acute cellular rejection (grade 2), for which patient #1 and patient #2 received treatments with high doses of steroids. Table 4 shows the post-LTx features.

**Table 4.** Features of post-transplantation period

Patient	# 1	# 2	# 3
Induction therapy	Methylprednisolone	Basiliximab + methylprednisolone	Basiliximab + methylprednisolone
Postoperative prolonged ECMO	No	No	No
PGD at 72 hours (grade)	0	2	0
Length of mechanical ventilation (days)	6	11	6
Length of stay in ICU (days)	15	47	7
Bleeding requiring chest reopening	No	No	No
Critical illness neuropathy	Yes	Yes	Yes
Complicated pleural effusion	No	No	Yes
Overall survival	Dead	Dead	Alive
Length of survival (days)	47	52	30
Discharge	No	No	Yes

ECMO = extracorporeal membrane oxygenation; PGD = primary graft dysfunction; ICU = intensive care unit.

## DISCUSSION

The world is currently living through the worst pandemic in human history, with more than four million deaths due to COVID-19. There has been widespread discussion of how to prevent and treat COVID-19. In this manner, LTx has become a treatment option for patients with severe ARDS and irreversible pulmonary fibrosis. Nonetheless, the outcomes remain unclear and few data are yet available in the international literature. Patients with severe COVID-19 are critically ill and develop considerable degrees of ICU-related comorbidities at the time that lung transplantation is considered.^11^

When LTx as a treatment for acute illnesses like COVID-19 has been discussed with lung transplantation teams, several ethical questions have been raised.^12^ During the pandemic, there has been a reduction in the offer of donors, and procurement teams have had to be more prudent because of the risk of donor-derived infection, given that there are great numbers of COVID-19 asymptomatic patients.^13^^,^^14^

Cypel and Keshavjee recommended that transplantation centers should have access to a broad donor pool and would need to have low waiting-list mortality. This will maintain fair and equitable donor organ allocation and provide the chance of life-saving organ transplantation for patients who are more likely to survive.^12^ On the other hand, in Brazil, there is no organ allocation score, and the waiting list is generated in accordance with entry time. Therefore, every case of prioritization needs to receive approval from the Technical Committee for Thoracic Organ Transplantation of the state of São Paulo.

Use of telemedicine has grown during the pandemic for several reasons. Our team uses telemedicine to screen cases from other centers, since we are a public/private reference service.^15^ Up to the present time, we have discussed the cases of five patients via telemedicine, but only one of them met the criteria. Fortunately, she recovered partially and presented improved lung function. Telemedicine is an important tool in a reference center. Our hospital has started a TELE-ICU service to discuss difficult cases and help physicians anywhere in Brazil to better manage critically ill COVID-19 patients.^16^

The surgical strategy that we use to perform LTx is similar to the one published by the Vienna Group,^10^ with a central VA ECMO circuit installed. The VV ECMO was kept running in parallel with a reduced flow of 1.5 liters/minute, while the Vienna Group reduced the flow to 1.0 liters/minute. This tactic allowed greater security throughout the procedures. We emphasize that dissection during native lung pneumonectomy is challenging. Dense pleuropulmonary adhesions were found in case #1 although both cases had pleural drainage. We found highly vascularized and thickened mediastinal and parietal pleura. Furthermore, bulky pulmonary hilar lymphadenopathy was encountered in all cases.^11^

Our results were similar to those of other groups.^9^^,^^10^^,^^11^^,^^17^ After the first cases were published, Cypel and Keshavjee suggested in Lancet Respiratory that recipient selection should be done on the basis of ratifying previous reports, with all ethical considerations.^12^ The study that forms the largest publication so far was conducted in accordance with these same considerations.^11^

## CONCLUSIONS

This report represents our initial experience with LTx after severe COVID-19, in Brazil. Unfortunately, data remain scarce, as also seen in this study, especially regarding information about long-term outcomes. However, SARS-COV-2 continues to infect hundreds of thousands of people worldwide each day and LTx will always be an option for saving patients with severe COVID-19. Our report showed that LTx is feasible among these complex patients. Survival over the first 30 days was 100%, and this favors surgical feasibility. Nonetheless, these were critically ill patients. We recommend that a multidisciplinary team with institutional engagement should be assembled for such cases. Patients presenting end-stage lung disease alone who are undergoing rehabilitation and are awake can be considered for transplantation. If these patients’ conditions deteriorate or improve, they should be withdrawn from the waitlist. For this reason, implementation of a concomitant palliative care approach is fundamental.

## Appendix

**Appendix 1.** Regarding eligibility for evaluation and listing for lung transplantation, the recipient patients must fulfill all the requirements, as follows:

Negative SARS-CoV-2 RT-PCR tests - minimum of 2 tests within an interval of 24-48 hours and at least one of the samples needs to be from the inferior respiratory tract (BAL, sputum or tracheal aspirate). Age < 65 years. The transplant center has independence to decide its maximum age limit for lung transplantation, but it must not exceed 65 years. Considering the ISHLT guidelines regarding overall survival, the best outcomes are for recipients < 50 years old (ideal recipients ranging from 18 to 50 years). Out-of-range recipients may be evaluated, but listing will require deliberation by the Technical Chamber of the state of São Paulo. Irreversible pulmonary status, at least 6 weeks after symptoms onset, entailing dependency on invasive mechanical ventilation (24 hours/day) or ECMO > 4 weeks. Body mass index (BMI) ranging from 17 to 27 prior to hospitalization. Hemodynamic stability. Ideally, the recipient should have only one organic dysfunction, and should be able to maintain hemodynamic stability without the need for vasoactive drugs. Absence of active fungal or bacterial infection. Ideally, with the aim of ensuring success for the procedure, the recipient should not present uncontrolled infection complications or use of antimicrobial drugs at the time of listing or prioritization. Multi-drug resistant germ colonization is considered to be a risk factor. Patients need to be awake and agree to lung transplantation; they must understand the need for and the process of evaluation for listing. Caregivers must be assessed by psychologists and nursing teams. Social worker evaluation and approval is required. There needs to have been an absence of history of active smoking prior to the episode of COVID-19. Critically ill neuropathy will be tolerated, provided that there is at least grade 3 motor strength and it is possible to maintain rehabilitation while waiting for the organ. Left ventricle ejection fraction > 50%. Transesophageal echocardiogram without vegetations and no anatomical or functional abnormalities. Left cardiac catheterization without coronary obstructions that cannot be treated percutaneously, for any patient older than 50 years, as long as they have not had recent catheterization or normal coronary CT angiography in the last 2 years. For patients between 40 and 50 years of age, unobstructed coronary angiotomography is required. Pulmonary artery CT angiography is required for all ages. Absence of other chronic or irreversible organ dysfunctions. Absence of other acute disorders, including renal replacement therapy. Justification/reference: the criteria for inclusion on the waiting list need to encompass a creatinine clearance rate greater than 40 ml/min. Signing of the consent form for lung transplantation by a first-degree relative or spouse. The transplantation team has the autonomy to contraindicate the transplantation according to its judgment of the set of clinical information and the evaluation by the multidisciplinary team, regardless of the perception of other teams that provide care for the patient.
